# Acknowledgment to Reviewers of *Antibiotics* in 2021

**DOI:** 10.3390/antibiotics11020214

**Published:** 2022-02-08

**Authors:** Antibiotics Editorial Office

**Affiliations:** MDPI AG, St. Alban-Anlage 66, 4052 Basel, Switzerland

Rigorous peer-reviews are the basis of high-quality academic publishing. Thanks to the great efforts of our reviewers, *Antibiotics* was able to maintain its standards for the high quality of its published papers. Thanks to the contribution of our reviewers, in 2021, the median time to first decision was 14 days and the median time to publication was 35 days. The editors would like to extend their gratitude and recognition to the following reviewers for their precious time and dedication, regardless of whether the papers they reviewed were finally published:
Abasse, SoumethLedesma, Ana EstelaAbbasi Oshaghi, EbrahimLee, Cheng-IAbdel-Glil, MostafaLee, Ching-ChiAbd-Elsalam, Kamel AhmedLee, ChowAbdul-Aziz, HafizLee, Hye-raAbia, Akebe Luther KingLee, HyunÁbrányi-Balogh, PéterLee, Je ChulAbugri, Daniel A.Lee, Jong BongAbuillan, WasimLefei, JiaoAcero, NuriaLegnani, ClaudioAcharya, Kamal RajLeitão, AnaAchiron, AsafLemercier, GillesAćimović, MilicaLenart-Boroń, AnnaAdamczyk-Woźniak, AgnieszkaLenhard, Justin R.Adamek, MartinaLeon, FranciscoAdams, Sandra D.León, FranciscoAdebiyi, AdebowaleLeskovac, Andreja R.Adégbitè, Bayodé RoméoLesouhaitier, OlivierAdembri, ChiaraLesyk, RomanAdil, MohdLesyk, Roman B.Adnan, MohdLetek, MichalAgatemor, ChristianLevy, HaimAghajanyan, AnushLewandowicz, JacekAgoro, RafiouLi, BingAgresta, FerdinandoLi, CarmenAgudelo-Romero, PatriciaLi, GangAguirre-Álvarez, GabrielLi, Guang-LinAhmad, ShujaatLi, Guo-FuAhmad, Syed SayeedLi, JiyunAhmed, Mohamed Bedair M.Li, LuAlafiatayo, RuthLi, RuichaoAl-Ahmad, AliLiao, Shu-ChuanAlam, AsrafulLibera, KacperAlbarillo, Fritzie S.Licata, FrancescaAlbericio, FernandoLiebman, MichaelAlbuquerque, PatríciaLiechti, George W.Alcorn, SeanLifschitz, CarlosAldeyab, MamoonLim, Lyn LiAldred, KatieLima, BeatrizAlduina, RosaLin, Chuen-FuAlex, AnoopLin, HaiAlexander, GalushkoLin, HaishuAlfaro-Viquez, EmiliaLinares-Pastén, Javier A.Al-Fatimi, MohamedLinciano, PasqualeAlfonso, ZecconiLindahl, LasseAl-Hasan, Majdi N.Lindbæk, MortenAli, Mir MohammadLinden, SaraAlkan, MichaelLiou, Je-WenAl-Karmalawy, Ahmed A.Lipari, DarioAllegaert, KarelLipman, JeffreyAl-Maharik, NawafLiptáková, AdriánaAlmansoori, Akram AbdoLitowczenko, JagodaAlmeida, Anabela M.Liu, ChenxiAlqahtani, FalehLiu, Cheuk LunAlves-Silva, Jorge M.Liu, Hai-yangAmagliani, GiuliaLiu, JunyanAmils, RicardoLiu, XiaomeiAmmendola, SerenaLlama-Palacios, AranchaAnagnostakos, KonstantinosLlena, Carmen C.Anastasopoulos, EleftheriosLlor, CarlAnastasopoulou, AmaliaLobato Tapia, Carlos AlbertoAncuceanu, RobertLøbner-Olesen, AndersAnda, BaicusŁochyńska, MałgorzataAndrianasolo, EricLoontjens, Ton J. A.Ang, Geik YongLopatkin, Allison J.Angelici, Maria CristinaLopes, GraçaAngelov, BorislavLópez Garcia, José ManuelAngelova, SilviaLópez, CeferinoAnsari, Mohd NazamLopez-Cortes, LuisAnstead, Gregory M.Lopez-Jornet, PiaAntonelli, AlbertoLora-Tamayo, JaimeAntunes, JoanaLorenzutti, Augusto MatíasAntunes, JorgeLounsbury, NicoleAnyanwu, PhilipLožienė, KristinaAoki, KotaroLozniewski, AlainAqib, Amjad IslamLu, ShaoyongArafat, NagahLucchetti, JacopoArana, InesLui, GraceArchbald-Pannone, LaurieŁukaszewski, MarceliAremu, TaiwoLunge, Vagner RicardoAriyoshi, NobuhiroLuong, MinhArmalyte, JulijaLuppi, BarbaraArmengol, RamónLupu, DianaArteaga-Livias, KovyLuqman, ArifArukha, Ananta PrasadLurie-Weinberger, Mor NadiaArunachalam, KannappanLusardi, KatherineArunasri, KotakondaLuther King, Abia AkebeAshbrook, Aaron R.Lysnyansky, InnaAshour, HossamMa, Lay SunAshour, Mohamed LotfyMaamar, ElaaAshraf, Syed AmirMacabeo, Allan Patrick G.Aspatwar, AshokMachado, AnaAsquith, Christopher R. M.Machado, JorgeAssiri, Abdullah A.Machado, RaulAtique, UsmanMacori, GuerrinoAtobe, MasakazuMad′ar, MariánAtrián-Blasco, ElenaMadariaga, LeyreAttia, Ahmed SherifMaddox, CarolAttiq Rehman, MuhammadMadkour, MahmoudAudette, Gerald F.Maggi, FilippoAudia, Jonathon P.Magiorkinis, EmmanouilAugustine, Swinburne A. J.Maguin, EmmanuelleAugustyniak, AdrianMahmoud, AymanAujayeb, AvinashMahmoudvand, HosseinAung, Kyaw ThuMai, Hang NgaAuriti, CinziaMaisano, DomenicoAvedissian, Sean N.Maisetta, GiuseppantonioAvishai, GalMajkowska-Skrobek, GrażynaAwad, MilenaMajtan, JurajAwasthi, MayankaMajumdar, ManasiAwatade, NikhilMakarewicz, OliwiaAwosile, BabafelaMalandrakis, EmmanouilAxiotis, EvangelosMalcangi, GiuseppinaAyala, JuanMaldonado-Carmona, NidiaAydin, MalikMalla, BikashAyub, RabiaMallah, NarmeenAzevedo, Nuno F.Malm, AnnaAzrad, MayaMaloy, StanleyBabicz, MarekMamani, Luis Daniel GoyzuetaBabtan, Anida MariaMan, AdrianBach, HoracioMangiaterra, GianmarcoBagattini, MariaManicone, Paolo FrancescoBagheri, HamidrezaManolescu, Loredana Sabina CorneliaBagiu, Iulia-CristinaManuel, CandidaBaindara, PiyushMarabotti, AnnaBaio, RaffaeleMaraolo, Alberto EnricoBaldisserotto, AnnaMarcelino, PauloBaldy-Chudzik, KatarzynaMarchel, ŁukaszBalhaddad, Abdulrahman A.Mare, AncaBalin, KatarzynaMareković, IvanaBalkanska, RalitsaMaria De LourdesBanche, GiulianaMariano, FilippoBanerjee, KasturiMarincu, IosifBaniahmad, AriaMarino, Anna M. F.Banks, LoriMarino, GabrieleBaptista, RafaelMarkovska, RumyanaBaralla, ElenaMarongiu, GiuseppeBarantsevich, E. P.Marotta, ClaudiaBarata, PedroMarques, CátiaBaratella, ElisaMarsili, EnricoBarausse, CarloMartín, Juan FranciscoBarbieri, ElisaMartinez Rodriguez, FlemingBarchitta, MartinaMartínez, José LuisBardají, EduardMartínez-Águila, AlejandroBari, EliaMartínez-Martínez, SoniaBar-Joseph, MosheMartínez-Morales, FernandoBarnard, Ross T.Martin-Gomez, M. TeresaBarquero-Calvo, ElíasMartini, CeciliaBarragan, Adrian A.Martini, FlorenciaBarrasa, HelenaMartino, Piera AnnaBarreca, DavideMartín-Quirós, AlejandroBarreca, SalvatoreMartins, NataliaBarreto, HumbertoMartins, NatáliaBartel, JürgenMarto, Carlos MiguelBartosik, Aneta AgnieszkaMartorana, Alessandra M.Basco, LeonardoMartu, Maria-AlexandraBassolé, Imaël Henri NestorMaruca, AnnalisaBatinovic, StevenMarutescu, Luminita GabrielaBatzias, GeorgiosMarzuillo, CarolinaBaumann, MarcusMascellino, Maria TeresaBavaro, DavideMasci, Valentina LaghezzaBecker, KarstenMashima, IzumiBehzadi, PayamMaslanova, IvanaBeld, JorisMatei, CristianBellance, NadègeMathew, BasilBelli, Carla BargiMathurkar, ShashwatiBello Toledo, HeliaMatsuda, AkiraBencúrová, ElenaMattila, SariBendaoud, MeriemMattoo, AutarBendary, MahmoudMatuszewska, MartaBenedetti, FrancescaMatuz, MariaBenetti, GiulioMauritzen, Jesper JuelBéni, SzabolcsMauro, Maria VittoriaBenini, AnnaMazurek-Popczyk, JustynaBenito, Juan M.Mazzeo, Paolo PioBenito, MónicaMazzitelli, MariaBennett, John E.Mazzotta, SarahBentancor, Leticia V.Mboera, Leonard E. G.Benton, AngelaMboowa, GeraldBerge, CatharinaMcgee, David J.Bergh, ØivindMcLucas, BruceBeri, DivyaMcMillan, Christopher L. D.Berkes, CharlotteMcVicker, GarethBerlov, MikhailMd. Aminul, IslamBernal, EvaMedaglia, ChiaraBernardini, GiuliaMeesters, KevinBernards, Alexandra T.Mehbub, Mohammad FerdousBertelloni, FabrizioMehra, KashishBerthaud, RomainMeini, Maria-RocioBerthold-Pluta, AnnaMeisel, LeonorBerti, Andrew DavidMejía-Ruíz, Claudio HumbertoBerzins, AivarsMenanteau–Ledouble, SimonBettache, NadirMendes, Marta V.Bettridge, JudyMendes, RafaelBevilacqua, AntonioMercer, Derry K.Bezirtzoglou, EugeniaMergoni, GiovanniBhagavathula, AkshayaMerola, CarmineBhatt, PankajMeroni, GabrielleBhattacharya, SomanonMesa, FranciscoBhattacharyya, SanchariMeschiari, MariannaBi, YangMeshram, ChetanBian, XiaoyingMesquita, CristinaBicker, JoanaMestres, ConxitaBielenica, AnnaMeto, AidaBientinesi, RiccardoMeybeck, AgnèsBiernasiuk, AnnaMeyers, Ian A.Biernat, Monika MariaMichalska-Sionkowska, MartaBigot, SarahMihelčić, MirnaBijle, Mohammed NadeemMiklasińska-Majdanik, MariaBinda, ElisaMilanova, AneliyaBinti Kamaruzzaman, Nor FadhilahMilewski, SlawomirBírošová, LuciaMin, Won LeeBisceglie, FrancoMinotti, ChiaraBjörkman, IngeborgMiranda, AdelaideBläckberg, AnnaMiranda, CarlaBlackwell, BarbaraMiranda-Sanchez, FabiolaBlagojevic Zagorac, GordanaMirete, SalvadorBlaha, ThomasMirijello, AntonioBlanco, Kate CristinaMirmozaffari, MirpouyaBlasco, LuciaMirón-Canelo, José-AntonioBlasdel, BobMishra, AbhishekBlazevic, IvicaMishra, NigamBleibtreu, AlexandreMisseri, GiovanniBloemberg, GuidoMitra, AvishekBocchinfuso, GianfrancoMittelmeier, WolframBochniarz, MariolaMiyamoto, HirotakaBockmühl, DirkMlaga, Kodjovi D.Boels, DavidMnif, BasamaBogossian, ElisaMnisi, Caven MguvaneBohn, ErwinMoawad, AmiraBoiko, IrynaMobashery, ShahriarBojinov, BojinModun, DarkoBok, EwaMofidfar, MohammadBokhary, HamidMognetti, BarbaraBoldizsár, ImreMohammed, AkramBoldura, Oana-MariaMohan, GaneshBonah, ErnestMohanram, HariniBonciu, ElenaMohsin, MashkoorBondžić, Aleksandra M.Mojica, MariaBongiorno, DafneMojsoska, BiljanaBonvicini, FrancescaMoloney, Mark G.Bookstaver, P. BrandonMomčilović, Stefan D.Borges, AnabelaMonchi, MehranBori, GuillemMondal, GoutamBork, Jacqueline T.Monroy, FernandoBoroń-Kaczmarska, AnnaMonteiro, CarlosBoschetti, GiovanniMonteiro, SilviaBose, Rajendran J. C.Moon, YuseokBostik, PavelMorak-Młodawska, BeataBottagisio, MartaMorandi, MattiaBouchami, OnsMore, ShyamBowden, BruceMorel, ChantalBoyen, FilipMorency-Potvin, PhilippeBozic, JoskoMoreno Switt, AndreaBozzo, GiancarloMoreno, Andrea MickeBraconi, DanielaMoreno, M. A.Brancaccio, MariaritaMorgovan, ClaudiuBranco, PatríciaMorikawa, KazuyaBratton, BenjaminMorita, YujiBrattsand, MariaMoro, GiuliaBrecker, LotharMorris, Andrew ConwayBrezoiu, Ana-MariaMorroni, GianlicaBrichacek, MatthewMorze, JakubBright, RichardMossialos, DimitrisBritz, Margaret L.Mot, AugustinBrotons-Canto, AnaMourenza, ÁlvaroBrukner, IvanMowlaboccus, ShakeelBruno Luigi Rocchi, MarcoMoyá, María LuisaBubnov, RostyslavMuchembled, JeromeBuchanan, RobertMuciño, JimenaBuchovec, IrinaMugetti, DavideBueno-Silva, BrunoMuhvic Urek, MirandaBugarin, AlejandroMukherjee, DwaipayanBujdoso, GezaMukherjee, MaitreyeeBukić, JosipaMulcahy, Ellyn R.Bulet, PhilippeMuller, CécileBunev, AlexanderMúñez, ElenaBungau, SimonaMuntean, DeliaBurchacka, EwaMunteanu, Florentina-DanielaBurgos-Ramos, EmmaMur, IsabelBurokiene, DaivaMurias, MarekBursic, VojislavaMurphy, AndrewBusila, MarianaMurri, RitaBustos-Martínez, JaimeMusoke, DavidButera, AndreaMusso, LoanaButler, DavidMussury, Rosilda M.Buttimer, ColinMuth, GüntherByun, Soo-HwanNaber, KurtCabal Rosel, AdrianaNadai, ChiaraCabrefiga, JordiNadǎş, George CosminCacciola, FrancescoNafis, AhmedCadelis, Melissa MichelleNagy, BelaCaeiro, Maria FilomenaNahum, AmitCalabrese, Francesco MariaNair, Anroop B.Calcagnile, MatteoNakakido, MakotoCalcutt, MichaelNale, JanetCamacho-Alonso, FabioNanda, SatyabrataCameron, DanielNanno, MasanobuCameron, David RobertNante, NicolaCampoccia, DavideNapolitano, FrancescoCampos, JoanaNarajczyk, MagdalenaCampos, Maria Jorge GeraldesNarita, MitsuoCaneiras, CátiaNasreen, SharifaCannon, RichardNatale, PaoloCapparè, PaoloNavalkar, KrupaCarabetta, Valerie J.Navarro-Alvarez, NaluCarattoli, AlessandraNavas, JesúsCarissimi, FrancescaNawrot-Hadzik, IzabelaCarmona-Ribeiro, Ana M.Nazik, HasanCarone, Benjamin R.Neelakantan, PrasannaCarotenuto, AlfonsoNegi, ArvindCarpinella, CeciliaNegut, IrinaCarretto, EdoardoNemec, AlexandrCarrillo, JenifferNesher, LiorCarta, PaolaNesterkina, MariiaCascio, AntonioNeureiter, DanielCasertano, MarcelloNeut, ChristelCasjens, SherwoodNguyen, LeeCassini, AlessandroNguyen, Minh-ThuCastagna, FabioNguyen, ScottCastaneda, ClaudiaNi, KaiyuanCastellani, DanieleNica, DianaCastilho, PaulaNica, Luminiţa MariaCastro Alves, RicardoNicholas, Ekow ThomfordCatalano, AlessiaNicholas, RobinCatry, BoudewijnNicholson, Ainsley C.Cazorla-Luna, RaúlNicolai, EleonoraCelenza, GiuseppeNicosia, AldoCenci-Goga, Beniamino T.Niculae, MihaelaCerbu, ConstantinNiemczak, MichalČernáková, LuciaNiestępski, SebastianCervino, GabrieleNieto, Teresa PérezChaber, RadosławNiewczas, Agata M.Chai, LilongNikolay, RainerChai, Tsun-ThaiNishida, SatoshiChallagundla, LavanyaNiu, ZhongweiChamakura, Karthik R.Nomoto, RyoheiChamieh, AmandaNorbrega, FranklinChandrasekaran, MurugesanNørskov-Lauritsen, NielsChang, Geng-RueiNosotti, Maria GiuliaChang, Wen-WeiNovak, JuricaCharani, EsmitaNovelli, GiorgioCharvat, RobertNovikov, AlexanderChassot, FrancieliNowak, AnnaChatterjee, Som S.Nowak, BeataChaudhri, VirendraNowakiewicz, AnetaChavez-Galan, LeslieNucci, LudovicaChen, ChaolingNulens, EricChen, ChenNunes, MonicaChen, FengjuiNurjadi, DennisChen, Jen-YinO′Loughlin, Colleen T.Chen, Lih-ChyangOchiuz, LacramioaraChen, Rong-JaneOćwieja, MagdalenaCheng, Hung-ChiOdoch, TerenceCheng, Jya-WeiOgawa, MasahiroCheng, ZishuoOgrodowczyk, AnnaChianese, AnnalisaOgunwande, Isiaka AjaniChiappim, WilliamOh, JaeseongChiavaroli, AnnalisaOishi, KenjiChien, Hsiu-WenOka, KyokoChifiriuc, CarmenOla, Antonius R. B.Chifiriuc, Mariana CarmenOlejnik, MalgorzataChilambi, Gayatri ShankarOliveira, Guedmiller S.Chin, Alex Wing HongOliveira, HugoChiou, Chien-ShunOloso, Nurudeen OlalekanChirico, GaetanoOlson, LinusChiş, Adriana AureliaOnorato, LorenzoChiu, Chia-YuOnyedibe, Kenneth I.Cho, SohyunOppliger, AnneChoi, Kyu YoungOpydo-Szymaczek, JustynaChoi, Peter J.Oriol, IsabelCholewinski, GrzegorzOrkusz, AgnieszkaChomicz, LidiaOrlando, GabriellaChong, Yong PilOrlić, SandiChow, Franklin Wang-ngaiOrsini, MassimilianoChrysanthakopoulos, Nikolaos A.Osivand, AsmaChu, ChishihOsonga, Francis J.Chung, Eun KyoungOsório, Nuno S.Chupakhin, EvgenyOsterman, Ilya A.Chwalibog, AndréOzaltin, KadirChyan, Chia- LinPachón, JerónimoCiacci, NagaiaPaczkowska, AnnaCilia, GiovanniPaczkowski, JonCiliberti, Maria GiovannaPadnya, PavelCinteza, OtiliaPadoin, ChristopheCitarasu, ThavasimuthuPadrão, JorgeCiura, KrzesimirPagani, LeonardoČižman, MilanPagniez, FabriceClark, CliffordPailhoriès, HélèneClarke, AnthonyPais, GwendolynClegg, SimonPakbin, BabakCobo, JavierPal, GargiCoelho, AnaPál, LászlóCojutti, Pier GiorgioPalagano, EleonoraColeman, Anthony WilliamPalagin, IvanColliers, AnneliesPallotto, CarloColonna, BiancaPalma Sircili, MarceloComan, CristianPalma, Paulo J.Contaldo, MariaPan, Po-shenCopolovici, Dana MariaPanderi, IreneCornea-Cipcigan, MihaielaPandur, EdinaCorona, AlbertoPant, AmitCorral-Gudino, LuisPantanella, FabrizioCorrò, MichelaPantel, AlixCortegiani, AndreaPanzarasa, GuidoCorte-Real, AnaPapadopoulos, GeorgiosCortés, Maria PilarPapaianni, MarinaCosgarea, RalucaPaparella, Ashleigh S.Cosmin Mihai, VesaPapi, PieroCotella, DiegoPapp-Wallace, Krisztina M.Cozza, GiorgioParada, RodolfoCraine, NoelParafati, LuciaCremers, Niels A. J.Paramythiotou, ElizabethCrespo-Rivas, Juan CarlosParente, EugenioCriscuolo, MariannaPark, DanielCristache, Corina MarilenaPark, HyunCristina, Romeo T.Park, Il-KwonCrnkovic, AnaPark, Jae YongCrossman, LisaPark, Jun-BeomCruz, Rui M. S.Park, Se ChangCruz-Morales, PabloPark, YoungjinCubero Pablo, María JoséParrish II, Richard H.Culebras, EstherParthasarathy, AnutthamanCultrera, RosarioParvej, Md ShafiullahCunha, EvaPârvu, MarcelCunha, MariaPașca, ClaudiaCurrie, KayPashikanti, SrinathCzepiel, JacekPassero, FelipeCzernel, GrzegorzPatatanian, EdnaD′Accolti, MariaPatching, Simon G.D′Agostino, DonatoPatel, ChhayaDa Silva, Luís C. N.Paterová, PavlaDa Silva, Luis Cláudio NascimentoPatino-Navarrete, RafaelDabrowski, JanuszPatra, Jayanta KumarDalessandri, DomenicoPatrick, S. GellingsDandekar, ThomasPaudel, AtmikaD'Andrea, Marco MariaPavarina, Ana ClaudiaDanielak, DorotaPavlović, HrvojeDanilo, Belviso BennyPeckham, Gabriel D.Danne, CamillePecyna, PaulinaDansette, PatrickPellegrini, MarikaDanziger, Larry H.Pellegrino, GerardoD'Ardes, DamianoPelullo, Concetta PaolaDash, Ranjeet PrasadPenrith, Mary-LouiseDato, Guglielmo ActisPenttinen, ReettaDaugelavicius, RimantasPerchec Merien, Anne-MarieDavies, Anita A.Pereira, Andre C.Davolos, DomenicoPeretz, AviDayal, NeetuPérez-Blanco, JonásDe Abreu Filho, Benicio AlvesPérez-Prieto, DanielDe Almeida, José RafaelPérez-Surio, Alberto FrutosDe Aquino, Francisco José TôrresPericas, Juan ManuelDe Bellis, LuigiPerisin, AnaDe Biase, DanielaPermana, Andi DianDe Biasi, Margherita G.Peruzy, Maria FrancescaDe Briyne, NancyPesavento, PatriciaDe Carvalho Formiga, MárcioPessler, FrankDe Carvalho, Isabel Loreto BarrosoPetat, HortenseDe La Fe, ChristianPeter, MartinDe La Pena Atehortua, Nelson A.Peters, AndrewDe Lagarde, MaudPetříčková, KateřinaDe Lurdes Dapkevicius, MariaPetrović, Aleksandra P.De Marco, IolandaPetrovykh, Dmitri Y.De Martino, LuisaPhan, Chin-SoonDe Mets, FrançoisPhan, Thi Tuong VyDe Oliveira Lima, EdeltrudesPhilippe, CécileDe Plano, LauraPhogat, SanjayDe Souza, Danival JoséPieczonka, Adam M.De Vito, CorradoPiegza, MichałDe Zotti, MartaPieszka, MarekDea-Ayuela, MaríaPietrella, DonatellaDearborn, Altaira D.Pijls, BartDeb, SubrataPina-Perez, Maria ConsueloDec, MartaPinho, EvaDecker, Sebastian O.Pinho, Mariana GomesDeflorio, MichelePinto, GraçaDel Campo, RosaPinto, LorisDellinger, E. PatchenPinto, SandraDeme, PragneyaPiras, SandraDeochand, DineshPires, CarlaDepoorter, ElizaPires, JoãoDeshpande, GauraviPires, Regina HelenaDey, AbhishekPiszczek, PiotrDhar, NeerajPitarch, AidaDhokale, RanjeetPitt, MirandaDhowlaghar, NitinPizzuto, Maresca AttardDi Blasio, AlessiaPlacido, AntonioDi Cerbo, AlessandroPlaczkiewicz, JagodaDi Gennaro, FrancescoPławińska-Czarnak, JoannaDi Giacomo, SilviaPlesiat, PatrickDi Giorgio, RobertoPletzer, DanielDi Lodovico, SilviaPłotka, MagdalenaDi Maro, AntimoPlüss-Suard, CatherineDi Martino, GiuseppePobiega, KatarzynaDi Salle, AnnaPoddighe, DimitriDi Somma, AngelaPodlewska, SabinaDíaz Martínez, MargaritaPogány Simonová, MonikaDiaz-Amaya, SusanaPohlig, FlorianDieckmann, RalfPoirel, LaurentDiez, RaquelPollard, DanicaDima, CristianPollera, ClaudiaDimitriou, GabrielPonce-Vargas, MiguelDimitrova, LyudmilaPopa, MirceaDimopoulou, DimitraPopescu, Răzvan-IonuțDincheva, IvaylaPopoff, Michel R.Ding, Bao-JianPopov, AntonDinos, GeorgiosPoradowski, DominikDiogo, PatriciaPorcelli, FernandoDišlers, AndrisPorter, EdithDivakaruni, SrinivasPotaniec, BartłomiejDkhar, Hedwin KitdorlangPotrykus, MartaDługaszewska, JolantaPotter, BethDobrzańska, JoannaPourcel, ChristineDocobo-Pérez, F.Pozzi, CeciliaDolzhenko, Anton V.Prada, GuillermoDominguez, Juan CarlosPraena-Segovia, JuliaDomínguez-Álvarez, EnriquePraveschotinunt, PichetDonadu, MatthewPrêcheur, IsabelleDooley, JamesPrieto, Ana Maria SahagunDoore, Sarah M.Primon-Barros, MurielDordet Frisoni, EmiliePrincipe, LuigiDors, ArkadiuszPrior, JoãoDoub, JamesPripdeevech, PatchareeDoyle, Declan A.Prosen, HelenaDoytchinova, IriniProtonotariou, EfthymiaDraenert, RikaProuillac, CarolineDragostin, Oana MariaProvenzano, DanieleDrasar, Pavel B.Prpić, JelenaDreier, JürgPruess, Birgit M.Drlica, KarlPsevdos, GeorgeDrozdowska, DanutaPuchkov, EvgenyDS, Francisco JavierPugliese, NicolaDuarte, AidaPuiggalí, JordiDubey, Ashutosh P.Purdoiu, Robert CristianDubey, NileshkumarPurkrtova, SabinaDuda, Marcel MateiQin, HuaDuda-Madej, AnnaQuaglio, DeborahDudhipala, NarendarQuilez, JoseDuerden, MartinQuirino, AngelaDukić, WalterQureshi, Ambrina A.Dumache, RalucaRadcliff, Fiona JaneDumancas, GerardRadtke, AleksandraDumic, IgorRagusa, RosaliaDumke, RogerRahman, A. F. M. MotiurDuong, PhuRahman, AbdurDuplaga, MariuszRahman, HadiarDurai, PrasannavenkateshRahman, Md. KaisarDuran, Sue HudsonRahman, MominurDurán-Poveda, ManuelRai, NavneetDurham, SpencerRaina, SatishDutta, SanjuctaRaizman, RoseDutu, LigiaRaj Rijal, KomalDuval, Raphaël E.Rajagopalan, KrithikaDüzgüneş, NejatRajaiya, JayaDylag, MariuszRajaure, ManojEcheverría-Esnal, DanielRakov, Alexey V.Eggers, Christian H.Ramachandra, SrinivasEick, SigrunRamburrun, PoornimaEinkauf, Jeffrey D.Ramírez, Ana SofíaEjaz, HasanRamos, PawełEjidike, Ikechukwu P.Ramos-Vivas, JoséEkateriniadou, LoukiaRangel, AndréEkwanzala, Mutshiene DeogratiasRaraigh, Karen S.El Khalloufi, FatimaRashid, MamunElekhnawy, EngyRasmussen, SkyeElfvin, AndersRasool, MuhammadElgamoudi, BassamRatajczak, MagdalenaElkins, KellyRather, IrfanEllepola, KassapaRathore, AtulElliman, JenniferRaval, YashEllis, Mary KathrynRay, Anindya SundarElshafie, Hazem S.Rayamajhee, BinodElsohaby, IbrahimRazvan-Cosmin, PetcaEltit, FelipeRedaelli, SimoneElwakil, BassmaRedfern, JamesEmecen-Huja, PinarReffuveille, FanyEmilia, RiggiRehman, HabibEpelde, FranciscoRehman, Md TabishErben, MauricioReich, AdamErkmen, OsmanReisfeld, SharonEscudero-Sanchez, RosaRemmas, NikolaosEskin, MichaelReuter, KlausEspinel-Ingroff, AnaReva, OlegEspinosa-Gongora, CarmenRexhepi, ImenaEsposito, PasqualeReynoso, DavidEsteban, JaimeRibeiro De Souza Da Cunha,Esteban-Cuesta, IreneRibeiro-Filho, JaimeEveillard, MatthieuRiccio, RaffaeleFacey, PaulRichert, AgnieszkaFajfr, MiroslavRichter, DanielFallarini, SilviaRico Yuste, AlbertoFaraoni, PaolaRiehm, JuliaFasina, FolorunsoRiggi, EmiliaFassouane, AzizRighi, EldaFazekašová, DanicaRipabelli, GiancarloFedorowicz, JoannaRiska, PaulFedorowicz, Joanna DagmaraRivera, DácilFelgueiras, HelenaRoby, Mohamed Hussein HamdyFelice, RobertoRoca-Millan, ElisabetFeliciano, JoanaRochfort, KeithFerguson, GayleRodgers, AoifeFerguson, RobertRodrigo, LuisFernández-Campos, FranciscoRodrigues Reis, Cristiano E.Fernández-Carrión, RebecaRodríguez Alfonso, Armando A.Fernandez-Garcia, RaquelRodríguez Díaz, Juan CarlosFerorelli, DavideRodríguez-Calleja, Jose MaríaFerreira Da Silva, ClassiusRodríguez-Lozano, Francisco JavierFerreira, Ana CristinaRodríguez-Pardo, DolorsFerreira, MarceloRodríguez-Rojas, AlexandroFerreira, MarianaRoggero, AngelaFerrini, FrancescoRogovskyy, ArtemFidalgo, AlexandraRohaim, MohammedFierro, José FernandoRoi, CiprianFigueiral, Maria HelenaRoig, Francisco J.Figueiras, AdolfoRojas, FedericoFijan, SabinaRojo, Maria AngelesFin, AndreaRojo-Poveda, OlgaFink, BerndRokaya, DineshFinšgar, MatjažRokney, AssafFiołka, Marta J.Rolo, JoanaFiore, MarcoRoman-Junior, WalterFisher, Jed F.Romeo, RobertoFleming, AoifeRomero, AlbertoFodor, LászlóRomero-González, RobertoFong, KarenRomero-Uribe, GabrielaFontana, CeciliaRonning, DonaldFort, IsabelRoque, FátimaFoster, DerekRosales-Mayor, EdmundoFoster, Simon J.Rosana Rocha, BarrosFotou, KonstantinaRosenau, FrankFoucher, DanielRostas, Arpad MihaiFouladkhah, AliyarRostkowska, OlgaFounou, Raspail CarrelRostoker, GuyFraile, LorenzoRoth, Klaus EdgarFrancesca, MensitieriRoviello, Giovanni N.Francikowski, JacekRoy, RenèFrancino, OlgaRoy, SagarFranco, Carlos M.Różańska, AnnaFranco, DomenicoRozza, ArianeFrassanito, AntonellaRuan, ZhiFreddi, LucaRubtsova, MayaFredheim, Elizabeth G. AaragRump, KatharinaFreitas, AndreiaRusic, DorisFridman, GregoryRusso, AlessandroFriedrichs, AnetteRusso, AntonioFrija, LuísRusu, AlexandruFris, MeganRusu, AuraFrosini, SianRuta, LaviniaFrye, Jonathan G.Ružauskas, ModestasFu, ChengzhangRybczyńska-Tkaczyk, KamilaFuchs, LiorRzewuska, MagdalenaFukuda, AkiraSabir, ShekhFumagalli, LauraSadeghpour Heravi, FatemahFurneri, Pio MariaSadowy, EwaFurtak, KarolinaSaffian, Shamin MohdFusco, AlessandraSaha, Sajal K.Gabant, PhilippeSahgal, PranshuGabriella, OrlandoSaidenberg, AndréGaffney, Erin M.Saied, Essa M.Gaglione, RosaSaijo, RyosukeGagliotti, CarloSakarikou, ChristinaGagyor, IldikoSaki, MortezaGajdács, MárióSalamon, DominikaGajdosik, Martina SrajerSalas, José BlancoGajewska, JoannaSaleh, MazenGalán-Sánchez, FátimaSalerno, CarloGalarce, NicolásSaliba Gustafsson, ErikaGaldiero, MassimilianoSalmeri, MarioGalindo, JohanSalo-Ahen, OutiGallo, GaetanoSalsano, AntonioGalluzzo, PaolaSalud, SerranoGamil, AmrSalvatore, PaolaGao, ChunxuSamal, SangramGao, JieSamardžija, MarkoGao, LiSampaio, AnaGarbacz, KatarzynaSamten, BukaGarbossa, Cesar Augusto PospissilSamuel, EssieGarcês, AndreiaSánchez, EmíliaGarcía, RodrigoSánchez-Hernández, EvaGarcia-Contreras, RodolfoSánchez-Montalvá, AdriánGarcía-Manrique, PabloSanders, James M.García-Muñoz Rodrigo, FerminSandra, MoralesGarcía-Quintanilla, MeritxellŞanlıbaba, PınarGarden, FrancesSantella, BiagioGarner, Bianca L.Santolalla-Arnedo, IvánGarus-Pakowska, AnnaSantos, JerranGatta, LuigiSapi, EvaGautier-Bouchardon, Anne-V.Saporiti, AlessandraGavrilovici, CristinaSaraiva, Raúl G.Gekenidis, Maria-TheresiaSaratale, Rijuta GaneshGelmini, FabrizioSaravanakumar, KandasamyGenovese, CarloSarkar, AmritaGerber, HannaSarría Santamera, AntonioGerdol, MarcoSartini, IreneGestal, MonicaSasahara, TeppeiGestal, Monica CartelleSatake, MasayukiGeszke-Moritz, MałgorzataSato, ChikashiGhasemian, AbdollahSavic, Ivan M.Ghenea, Alice ElenaSavitzky, AlanGhiciuc, Cristina MihaelaSavoie, JeanGhosh, SantoshSawai, JunGiacobbe, Daniele RobertoSawanyawisuth, KittisakGiammanco, AnnaSayed, Ahmed M.Gianchecchi, ElenaSayed, Ibrahim M.Gibson, HazelSblano, SabinaGica, NicolaeSchmidt, MartinGillespie, DavidSchmitt, FelixGiordani, BarbaraSchmitz, IngeGiordano, PaolaSchnabel, Ronny M.Giorgi, GiorgioSchneider, Elena K.Giormezis, NikolaosSchüller, ChristophGiovannenze, FrancescaSchulthess, BettinaGirolami, FlaviaSchwaiger, KarinGisbert-Garzarán, MiguelSchwan, WilliamGkentzi, DespoinaSchwarz, EdwardGladstone, Primrose BerylSchwarz, LukasGlassman, Patrick M.Schwarz, PatrickGnat, SebastianScott, AlisonGobin, IvanaScrano, LauraGoh, ShanScribano, DanielaGolemi-Kotra, DasantilaScutera, SaraGomes Neto, Joao CarlosScuteri, DamianaGomes, NelsonSeabra, CatarinaGómez-Fidalgo, ErnestoSegala, FrancescoGómez-Ríos, DavidŠegvić Klarić, MajaGómez-Zorrilla, SilviaSeidavi, AlirezaGonkowski, SławomirSelim, SamyGonzalez Domenech, Carmen MariaSellars, JonathanGonzález, Concepción C.Seoane, RafaelGonzález-Fandos, María ElenaSeok, HyeriGonzalez-López, JesúsSequeda-Castañeda, Luis G.González-Santamaría, JoséSergeeva, OlgaGorasiya, SamirSerra, DiegoGornatti Churria, Carlos DanielSerra, PatríciaGotoh, KenjiSerrano, Dolores R.Govindan, PrasanthSerwecinska, LilianaGoyal, AshishŠešelja Perišin, AnaGoyal, RinkajSettem, Rajendra P.Grande Tovar, Carlos DavidSetzer, William N.Granić, MarkoSevilla, EloísaGranica, SebastianSevilla-Morán, BeatrizGrare, MarionSferrazza, GianlucaGrasa, LauraShaaban, Mona I.Grau, SantiagoShaffer, MarkGreber, KatarzynaShah, Neel B.Greco, GraziaShahin, KhalidGregova, GabrielaShaik, Mohammed RafiGrgurina, IngeborgShaikh, SibhghatullaGrishin, Alexander V.Sharafutdinov, IrshadGroll, AndreasShariat, NikkiGrudlewska-Buda, KatarzynaSharma, AmitGrygorcewicz, BartłomiejSharma, AtulGuard, JeanSharma, MeghaGuarnieri, GabriellaSharma, PoonamGuerre, PhilippeShave, StevenGuillen Fretes, Rosa MariaShei, Ren-JayGulla, KrishnaShekhar Singh, ShashiGülses, AydinShelenkov, AndreyGündüz, MiyaseShen, JiangchuanGupta, Chhedi LalShen, ZhenyuGupta, KajalShetty, AmithGurney, JamesShi, YunlongGustafsson, RasmusShigemura, KatsumiGutierrez, ClaudeShimono, NobuyukiGutiérrez-Martin, César B.Shimozako, Helio JunjiGutierrez-Naranjo, JoseShin, Jae HyunGuzman, FannyShinozaki, YukikoGwiazdowska, DanielaShort, BrynGwon, KihakShree, SonalHaas, OskarShrivastava, Saurabh RamBihariLalHafeez, RahilaShumilina, D. V.Hagen, FrickmannSiddiqui, ArifHagihara, MaoSiddiqui, Mohammad JamshedHagiwara, KatsuroSiddiqui, Muhammad FaisalHajra, AdrijaSidorenko, SergeyHall, Jeffrey W.Siegel, AmandaHamada, YukihiroSierra, Josep M.Hamamoto, HiroshiSierra, MatildeHammerl, Jens AndréSignoriello, AnnaritaHammoudi Halat, DalalSigusch, Bernd W.Hampp, NorbertSikora, KarolHan, Mei-LingSilas Fernandes, EtoHan, ZheŠilha, DavidHanania, MichelSilva, Marcelo SousaHanieh, Patrizia NadiaSilva, Nathalia Cristina CironeHanif, NovriyandiSilva-Herzog, EugeniaHao, JinsongŠíma, MartinHardies, Stephen C.Simecka, Jerry W.Harnisz, MonikaSimitzis, PanagiotisHartke, AxelSimões, João Carlos CaetanoHasegawa, MakotoSimon, PhilippHassan, Ahmed AbdelkhalekSimonetti, GiovannaHassan, MariamŠindlerová, LenkaHassan, Sherif T. S.Singer, GeorgHaukka, KaisaSingh Gautam, Amit KumarHayes, Sidney J.Singh, AtheeshaHazan, RonenSingh, Brij PalHe, ShoukuiSingh, Pradip K.Heczko, Piotr B.Singh, ReemaHedman, HaydenSingh, SanjayHeegaard, Peter M. H.Singh, SukhvinderHeffernan, AaronSinnott, Catherine J.Hegedűs, CsabaSinopoli, AlessandraHeisig, PeterSionov, RonitHeney, ClaireSistla, SrinivasHeng, Nicholas C. K.Sisto, FrancescaHeredero-Bermejo, IreneSivaev, Igor B.Hernández, ÁngelSizova, OlgaHernández-García, MartaSkalska-Kamińska, AgnieszkaHernández-León, AlejandroSkaradzińska, AnetaHibbing, MichaelSkenderidis, ProdromosHIjikata, AtsushiSkerlavaj, BarbaraHindra, HindraSkliros, DimitriosHirade, TomohiroSkoglund, ErikHirai, JunSlate, AnthonyHirakawa, HidetadaSmith, AndrewHleba, LukasSmith, NicholasHoelzle, Ludwig E.Snyder, Orman L.Hofer, Matthias D.Sobur, Md. AbdusHoffman, Paul S.Sohma, YoshiroHojat, LeilaSolano, FranciscoHolatko, JiriSoliman, AhmedHollmann, AxelSolís Sanchez, GonzaloHolm, AnneSommer, JuliaHolt, Scott M.Song, PingHomma, TetsuyaSong, ShuangHong, Bo-YoungSoo Ko, KwanHoogland, DominiqueSousa, CláudiaHoque Apu, EhsanulSousa, JoanaHorhat, DeliaSousa, NunoHorhat, Florin GeorgeSousa, Sérgio F.Hort, KrishnaSouwer, Ibo H.Horvatek Tomic, DanijelaŠovljanski, OljaHorwitz, JacobSpadaro, SavinoHosseini, Mohammad-SalarSpinu, MarinaHosseinkhani, FaridehSpoto, SilviaHrabar, JerkoSroka, RonaldHristov, Peter IvanovStączek, SylwiaHsu, Bing-MuStanford, KimHu, ChunStappen, IrisHu, XiaowenStarčič Erjavec, MarjancaHuang, Fu-ChenStarosta, RadosławHuang, LeiStasevych, MarynaHuber, Charlotte A.Stasiak-Różańska, LidiaHuck, OlivierStasiowski, Michał JanHugerth, Luisa WarchavchikStaudacher, Jonas JaromirHulak, NatašaSteckiewicz, KarolHunter, Robert P.Steczkiewicz, KamilHuppler, Anna R.Stefanini, IreneHurley, JamesSteinbach-Rankins, JillHüsing, AnikaStępień-Pyśniak, DagmaraHussein, MaythamSter, CélineHutinet, GeoffreySterniša, MetaHwaiz, RundkStewart, AdamIandolo, AlfredoStiemsma, LeahIanni, AndreaStilinović, NebojšaIbba, RobertaStingu, Catalina-SuzanaIbrahim, MahmoudStratford, Robert E.Ibrahim, Maria SalemStraub, ChristinaIde, KazukiStrbak, OliverIelciu, IrinaStrukova, Elena N.Ignatov, AlexanderStrychalski, JanuszIijima, NoriakiStrzemski, MaciejIkeda, MasayukiSturgill, MarcIlagan, Ma. Xenia G.Sturtevant, JoyIno, KosukeSu, Chih-ChiaIntra, JariSu, Shih-BinIonita, PetreSubramani Paranthaman, BalasubramaniIorio, RaffaeleSugita, NorikoIovino, FedericoSugiura, KatsuakiIribarren, José AntonioSulis, GiorgiaIrrgang, AlexandraSultana Rekha, RokeyaIshii, TakahiroSumbatyan, NataliaIskandar, KatiaSuperti, FabianaIslam, Mohammad TarequlSurmann, KristinIslam, RabiulSuzuki, HiroyukiIsola, GaetanoŠvedienė, JurgitaIvanova, Anastasia A.Svetlov, MaksimIzabela, Kern-ZdanowiczSvircev, Antonet M.Izquierdo-Bueno, InmaculadaSwatko-Ossor, MartaJaehne, Anja KathrinŚwierczek, ArturJaggavarapu, SiddharthSzacon, ElzbietaJagielska, ElżbietaSzczepankiewicz, BarbaraJaja, Ishmael FestusSzczerba-Turek, AnnaJakab, EndreSzekeres, EdinaJakaria, Md.Szpyrka, EwaJaki, BirgitSzulczyk, DanielJakob, Roman P.Szultka-Młyńska, MałgorzataJana, BimalSzweda, PiotrJańczewski, DominikSzymańska, GrażynaJang, Ji-HyunTadin, AntonijaJang, Jong-HwaTagliaro, IreneJankovic, MomciloTagliavia, MarcelloJankowski, JanTahar, RachidaJanowicz, AnnaTahrioui, AliJansåker, FilipTailhades, JulienJansen, ChristianTalapko, JasminkaJaradat, NidalTalreja, NeetuJaros, Sabina W.Tambella, Adolfo MariaJaswal, RajneeshTamburro, ManuelaJedelská, JarmilaTampa, MirceaJesudhasan, PalmyTan, Jeremy P. K.Jesudoss Chelladurai, JebaTanabe, MikioJeżewska-Frąckowiak, JoannaTandukar, SarmilaJia, BaoleiTang, Ri-YuanJiang, HongxiaTanui, Collins K.Jiang, Jhih-HangTardugno, RobertaJibran, RubinaTartaglia, Gianluca M.Jin, Hyung JongTavío, María M.Jobs, KatarzynaTchertanov, LubaJogl, GerwaldTecilla, PaoloJohansen, Matt D.Teh, Cindy Shuan JuJolly, AmberTeixeira, PilarJończyk-Matysiak, EwaTeleky, Bernadette EmokeJordao, LuisaTellez-Isaias, GuillermoJoshi, SameerTemviriyanukul, PiyaJouini, AhlemTeng, PengJoulain, DanielTenhagen, Bernd-AloisJovanović, MarinaTer Kuile, BennoJoyce, PaulTerkawi, Mohamad AlaaJozanović, MarijaTernák, GáborJuárez-Castelló, Carmelo A.Tesi, LorenzoJukič, MarkoTessarin, GiulioJuluri, AbhishekThaker, Maulik N.Jun, Se-RanThanki, Anisha MahendraKacaniova, MiroslavaThanna, SandeepKaczor-Urnanowicz, Karolina ElzbietaTharmalingam, NagendranKadam, AvinashThomas, JulieKahn, Nicolas CarlosThompson, Dorothea K.Kalafatovic, DanielaThongprayoon, CharatKalaitzakis, EmmanouilThulstrup, Peter W.Kaldhone, PravinTichaczek-Goska, DorotaKalejs, OskarsTikhe, Chinmay V.Kalesse, MarkusTimenetsky, JorgeKalluru, PolirajuTintino, SauloKalogirou, Andreas S.Tiscar, Pietro G.Kamal, Mohammad AmjadTit, Delia MirelaKamaszewski, MaciejTkadlec, JanKamble, ShyamTkavc, RokKamei, JunTodorov, SvetoslavKamila, BobrekTogawa, AtsushiKamysz, ElżbietaToma, Elena-AdelinaKandil, SaharTomas, AnaKaneko, GenTomassone, LauraKang, CongBaoTomaszewska, EwaKang, HeonjoongTomczyk, ŁukaszKang, JiheeTopolski, MariuszKang, Kyung PyoToro, CeciliaKang, SanghoonTorres Barcelo, ClaraKanna Reddy, Hariprasada R.Toscano-Garibay, Julia D.Kao, Cheng-YenTossi, AlessandroKaradjian, GregoryToutain, Pierre LouisKaragouni, Amalia D.Tovar, Carlos David GrandeKarasneh, ReemaTownsend, EleanorKardos, GáborTragiannidis, AthanasiosKarlović, ZoranTramper-Stranders, Gerdien A.Karlsson, ErikTrček, JanjaKarobari, Mohmed IsaqaliTrifan, AdrianaKaruppan, Mohan Kumar MuthuTriggiano, FrancescoKashman, YoelTripathi, AmitKasoju, NareshTripathi, JitendraKaspar, HeikeTripathi, Jitendra KumarKaspersen, HåkonTrouvelot, SophieKato, HideoTrucillo, PaoloKatsafadou, AngelikiTrzaskowski, BartoszKatsoris, PanagiotisTscheliessing, RupertKatuchova, JanaTseng, Ping-HuiKauer, JosephTsouknidas, AlexanderKaur, GagandeepTuchscherr, LorenaKaushik, PrashantTurner, R. BriggKavaliauskas, PovilasTvrdá, EvaKavita, KumariTworowski, DmitryKawalek, AdamTyurin, Anton P.Kawasaki, KiyonoriTzang, Bor-ShowKawase, MasamiUchiyama, KiyotakaKayumov, AiratUdoh, Arit EsioKazim, NoorUl-Hasan, SabahKebriaei, RaziehUlusoy, BeyzaKefleyesus, AmanielUno, ShunsukeKelarakis, AntoniosUrcan, Adriana CristinaKelesi, MarthaUrdahl, Anne MargreteKelly, DervlaValachová, KatarínaKempf, FlorentValenzuela, MiryamKenna, Dervla T. D.Valiakos, GeorgeKępa, MałgorzataValm, Alex M.Kerekes, GyorgyValoti, ErmannoKhalil, Ahmed Mohamed AliVamanu, EmanuelKhan, AbbasVan Bambeke, FrançoiseKhan, Aman UllahVan De Pol, Alma C.Khan, Faiz UllahVan Der Aar, PietKhan, Firdous A.Van Der Sande, MarianneKhan, MerajuddinVan Eerde, AndréKhanye, Setshaba D.Vanderpas, JeanKharaghani, DavoodVarghese, JothiKharat, Arun S.Varsaki, AthanasiaKharel, Madan K.Vasco Vidal, AldrinKhurram, MuhammadVasilchenko, AlexeyKiefer, DorotheeVásquez-Velásquez, DavidKieffer, NicolasVaz, Josiana A.Kieliszek, MarekVázquez-López, RosalinoKiga, KotaroVázquez-Ucha, Juan CarlosKikuchi, HaruhisaVeiga Dos Santos, MarcosKikuchi, ShogoVeiga, MarianaKikusato, MotoiVellinga, AkkeKim, ChoonVeloo, AlidaKim, ChunkiVerbanac, DonatellaKim, DokyunVercelli, CristinaKim, HoonVergadi, EleniKim, Hwa-JungVergunst, Annette C.Kim, Jae-SeokVersporten, AnnKim, Ji HyungVibet, Marie-AnneKim, JieunVictor, BrunoKim, Jung MinVieira Baptista, PedroKim, Ki-YoungVilar Ares, Maria J.Kim, Kwang-sunVilović, MarinoKim, Sin-youngVinti, LucianaKim, SoochongVisca, PaoloKinthada, LakshmanakumarVishnyakov, Innokentii E.Kioshima, Erika SekiVisweswaran, Ganesh RamKirchberger, PaulVlaicu, Ioana DorinaKiss, TivadarVon Zeska Kress, Márcia ReginaKitano, YoshikazuVoronina, O. L.Kittl, SonjaVujačić Nikezić, AnaKlar, AgnesVuletić, MarkoKlassen, RolandWalker, Robert J.Klausner, JeffreyWalsh, Catherine J.Kleczkowska, PatrycjaWalsh, FionaKlewicka, ElzbietaWalsh, Laurence J.Klibi, NaouelWalter, Jasmine A.Klobučar, HrvojeWang, Chao-MinKlyczek, KarenWang, Chung-ChengKnighton, ShaninaWang, DongdongKocot, AleksandraWang, GuangshunKocot, Aleksandra MariaWang, Jing-QuanKocsis, BelaWang, Jiun-LinKojic, MilanWang, Myeong-HyeonKokkinakis, EmmanouilWang, QiKokoska, LadislavWang, Shao-HungKolář, MilanWang, ShenqiKolar, MitjaWasfi, RehamKolobov, Alexandr AlexandrovichWatanabe, EvandroKolota, AleksandraWatanabe, JunKolpa, MalgorzataWawrzyniak, JolantaKolzunova, LidiiaWdowiak-Wróbel, SylwiaKomaki, HisayukiWeadge, JoelKonduri, SrihariWeaver, KeithKong, CongWegrzyn, AlicjaKonieczka, PawełWegrzyn, GrzegorzKonjevic, DeanWei, HongpingKonstantinidi, AikateriniWeigel, MarkusKontogiorgis, ChristosWeis, Allison M.Koolman, LeonardWerner, EnriqueKopsidas, IoannisWertz, Philip W.Kordek, AgnieszkaWieczor, MiloszKorf, ImkeWieczorek, DorotaKorla, Praveen KumarWiglusz, KatarzynaKorneev, DenisWiktorczyk-Kapischke, NataliaKornitzer, DanielWillett, JuliaKorona-Glowniak, IzabelaWilson, James W.Korzekwa, KamilaWinckel, Myriam VanKorzeniewska, EwaWinders, Hana RacKosecka-Strojek, MajaWiraja, ChristianKosnik, MitjaWójcicki, MichałKostelac, DeniWojciechowska, MonikaKot, BarbaraWójcik, AnnaKotey, Fleischer C. N.Wójcik-Pszczoła, KatarzynaKotha, Raghavendhar R.Wojtyczka, RobertKotlowski, RomanWollny, AnjaKoutelidakis, Antonios E.Wolna-Maruwka, AgnieszkaKovacs, RenatoWolny-Koładka, KatarzynaKovvasu, Surya PrakasaraoWorwag, MalgorzataKowalczyk, MagdalenaWozniakiewicz, MichalKowalczyk, PawełWróbel, AgnieszkaKoyack, MarcWu, LeiKozakiewicz, AnnaWu, Ming-ChengKozdrój, JacekWu, Whei-FenKozlov, RomanXaplanteri, PanagiotaKranz, AngelaXavier, Basil BrittoKranzler, MarkusXu, JianKrause, FrankYagüe, PaulaKrauze, MagdalenaYamamura, ShigeoKrawczyk, BeataYang, Chih-JenKrismer, BernhardYang, KunKrockow, EvaYasir, MuhammadKroeker, AndreaYeh, Kuang-ShengKrokidis, MariosYeung, Eugene Y. H.Kruszka, DariuszYilmaz, BahtiyarKrzyżek, PawełYilmaz, SevdanKubo, Anna-LiisaYoon, Eun-JeongKubo, MiwaYoon, WonsuckKucharíková, SoňaYu, Ollie YiruKucuk, IsrafilYu, ZiniuKuczyńska-Wiśnik, DorotaYukl, Erik T.Kuhn, AndreasZacconi, FlaviaKulakovskaya, Tatiana V.Zacharis, Constantinos K.Kulo Cesic, AidaZagaglia, CarloKumar, AnoopZaha, Dana CarmenKumar, HirdeshZahid, MuhammadKumar, ManishZakrocka, IzabelaKumar, NareshZaky, Ahmed A.Kumar, PradeepZalewska, MagdalenaKumar, RajZama, DanieleKumar, RupeshZandi, AshkanKumar, SandeepZannella, CarlaKumar, ShaileshZanolli Moreno, LuisaKumar, VikashZarfel, Gernot E.Kumari, PujaŻarowska, BarbaraKunicka-Styczyńska, AlinaZarrilli, RaffaeleKuo, HsiaohsuanZawada, KatarzynaKurekci, CemilZawadzka, KatarzynaKutkowska, JolantaZeeshan, MuhammadKwiatek, AgnieszkaZeineldin, MohamedKwiatkowski, PawelZeinhom, MohamedKwon, HyunZemb, OlivierKwon, JaeyoungZervas, AthanasiosLagatolla, CristinaZhang, ChunyeŁagowska, KarolinaZhang, ErshuaiŁagowski, DominikZhang, FanLai, Pin-KuangZhang, GuanshiLalak-Kańczugowska, JustynaZhang, JileiLamas, AlexandreZhang, LiqiangLancelin, Jean-MarcZhang, ShaozhongLanghansova, LenkaZhao, JinxinLányi, KatalinZhou, DongfangLapierre, LisetteZhu, DongLaskaris, ParisZieniuk, BartłomiejLaskowska, EwaZinigrad, MichaelLatter, SusanZnaidi, SadriLaudadio, EmilianoZobi, FabioLaudy, Agnieszka E.Zommiti, MohamedLaw, ChristopherZotti, FrancescaLaw, DavidZou, HuasongLazarev, Vassiliy N.Zubriene, AstaLe Questel, Jean-yvesŻurawska-Płaksej, Ewa

